# Effects of methimazole and propylthiouracil exposure during pregnancy on the risk of neonatal congenital malformations: A meta-analysis

**DOI:** 10.1371/journal.pone.0180108

**Published:** 2017-07-03

**Authors:** Rongjing Song, Hepu Lin, Yue Chen, Xiuying Zhang, Wanyu Feng

**Affiliations:** 1 Department of Pharmacy, Peking University People’s Hospital, Beijing, China; 2 Affiliated Bayi Brain Hospital, PLA Army General Hospital, Beijing, China; 3 Department of Endocrinology and Metabolism, Peking University People's Hospital, Beijing, China; Jilin University, CHINA

## Abstract

**Objective:**

The aim of this study was to determine the effect of exposure to different antithyroid drugs during pregnancy on the incidence of neonatal congenital malformations.

**Methods:**

A meta-analysis was performed to compare the incidence of neonatal congenital malformations after exposure to different antithyroid drugs during pregnancy. Twelve studies that met the inclusion criteria were included in this meta-analysis. PubMed, Embase, and CENTRAL databases were searched from inception until January 2017. Study designs included case–control studies, prospective cohort studies, and retrospective cohort studies.

**Results:**

Twelve studies involving 8028 participants with exposure to different antithyroid drugs during pregnancy were included in this study; however, only 10 studies involving 5059 participants involved exposure to different antithyroid drugs exactly during pregnancy. Our results indicated that exposure to methimazole (MMI)/carbimazole (CMZ) only during pregnancy significantly increased the risk of neonatal congenital malformations compared to no antithyroid drug exposure (OR 1.88; 95%CI 1.33 to 2.65; P = 0.0004). No differences were observed between propylthiouracil (PTU) exposure and no antithyroid drug exposure only during pregnancy (OR 0.81; 95%CI 0.58 to 1.15; P = 0.24). Exposure to MMI/CMZ only during pregnancy significantly increased the risk of neonatal congenital malformations compared to that associated with exposure to PTU (OR 1.90; 95%CI 1.30 to 2.78; P = 0.001).

**Conclusion:**

For pregnant women with hyperthyroidism, exposure to MMI/CMZ significantly increased the incidence of neonatal congenital malformations compared to exposure to PTU and no antithyroid drug exposure; however, no differences were observed between PTU exposure and no antithyroid drug exposure.

## Introduction

The prevalence of hyperthyroidism during pregnancy is approximately 0.1–0.2% [1]. Graves’ disease is the most common cause of gestational hyperthyroidism. In addition, other types of thyroid disorders, such as toxic multinodular goiter or solitary autonomously functioning nodules, induce gestational hyperthyroidism. Hyperthyroidism during pregnancy should be carefully treated because it can result in adverse maternal and neonatal outcomes. A cohort study performed by Mannisto et al. found that gestational hyperthyroidism was associated with an increased risk of labor induction, preeclampsia, superimposed preeclampsia, threatened and observed preterm births, and neonatal intensive care unit admission [2]. Therefore, there is a strong need to control hyperthyroidism during pregnancy.

Treatment options for hyperthyroidism include surgical treatment (partial or complete thyroidectomy), radioactive iodine, and antithyroid drugs (ATDs) [3]. Surgery and radioactive iodine therapy, however, are rarely used during pregnancy. Surgery should be reserved as the last line of treatment for the minority of severe cases of gestational hyperthyroidism and should only be performed during the second trimester of pregnancy. Radioactive iodine therapy is contraindicated during pregnancy because of the increased risk of subsequent fetal thyrotoxicosis [4]. Therefore, antithyroid agents, including methimazole (MMI)/carbimazole (CMZ) (pro-drug of methimazole) and propylthiouracil (PTU), have become the standard treatment for hyperthyroidism during pregnancy. However, their use is controversial owing to their adverse effects. MMI/CMZ can induce neonatal congenital malformations and PTU can induce maternal hepatotoxicity [3].

In recent years, new studies have indicated that PTU was associated with an increased risk of neonatal congenital anomalies [5, 6]. Chen’s research indicated that there was a higher risk of major congenital anomalies in the group exposed to PTU during pregnancy than in the group exposed to MMI [7]. In contrast, the results of other studies indicated conflicting conclusions [8]. Li et al. found that PTU was a safer choice for treating pregnant women with hyperthyroidism with respect to the risk of birth defects [9]. Moreover, several trials found no association between the use of ATDs in pregnancy and neonatal congenital malformations [10].

Therefore, the most appropriate agent for the management of hyperthyroidism during pregnancy with respect to the incidence of neonatal congenital malformations remains unclear. In this meta-analysis, we evaluated the risk of neonatal congenital malformations after exposure to different ATDs during pregnancy to reassess the effects of MMI and PTU exposure during pregnancy. We hope these findings provide new evidence for the management of gestational hyperthyroidism using the two ATDs.

## Materials and methods

**Fig 1:**
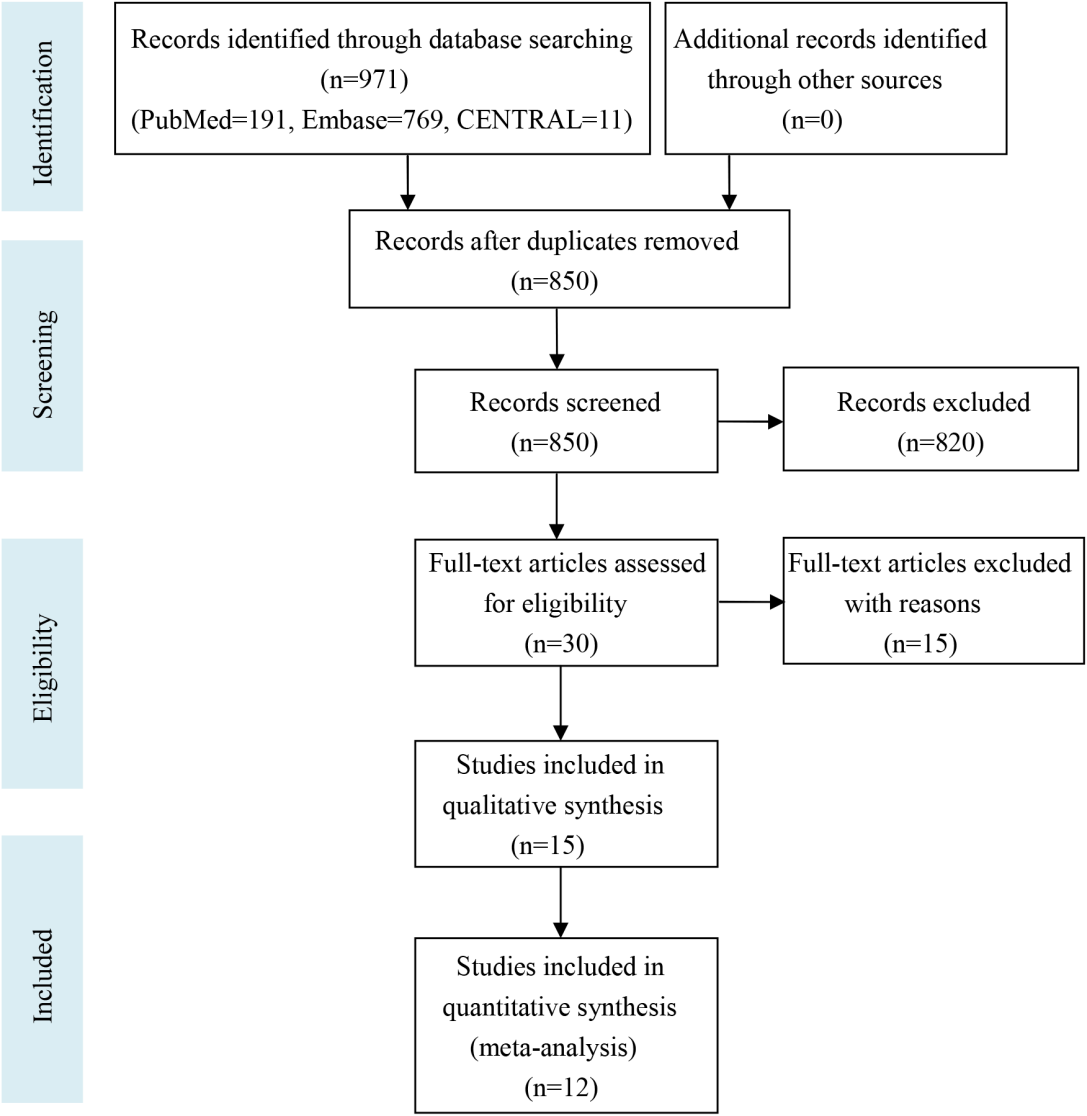
Flowchart of the study selection process for the meta-analysis.

Our meta-analysis follows the PRISMA (Preferred Reporting Items for Systematic Reviews and Meta-Analyses) guideline. The PRISMA flow diagram is shown in Fig 1 and the PRISMA checklist is shown in the S3 Table.

### Inclusion and exclusion criteria

The studies had to meet all the following criteria for inclusion: 1) subjects were pregnant women with hyperthyroidism who required antithyroid therapy; 2) subjects were administered MMI/CMZ and/or PTU; 3) neonatal congenital abnormalities, including any neonatal congenital abnormality or major neonatal congenital abnormalities, were presented as one or all of the outcomes in the study; 4) the study design was a case–control study, prospective cohort study, or retrospective cohort study. Studies published in English or Chinese and only studies with full text available were included in this meta-analysis. Case reports, systematic reviews, and studies without human data were excluded.

### Literature search

Three databases (PubMed, Embase, and the Cochrane library (Cochrane Central Register of Controlled Trials)) were searched using the following keywords from inception to January 2017: pregnancy; methimazole; propylthiouracil; carbimazole; antithyroid agents; congenital abnormalities; congenital defects; and congenital malformations. The search strategy is shown in the S1 Table.

### Study selection and data extraction

Rongjing Song and Hepu Lin independently performed the process of study selection and data collection. The following baseline data were collected for each study: authors, publication year, study design, study period, participants, exposure groups, no ATD group, control group, duration of exposure, and the type of congenital malformations. Disagreement was resolved by discussion with the third reviewer, Yue Chen.

### Quality assessment of the included studies

Two independent authors (Rongjing Song and Yue Chen) assessed the quality of each included study using the Newcastle-Ottawa Scale (NOS) system [11]. The “star system” has been developed in which a study is evaluated on three broad perspectives: the selection of the study groups; the comparability of the groups; and the ascertainment of either the exposure or outcome of interest for case–control or cohort studies, respectively. Eight items were included in the three perspectives (four items in selection, one item in comparability, and three items in outcome/exposure). A study can be awarded a maximum of one star for each numbered item within the selection and outcome/exposure categories. However, a maximum of two stars can be given for an item in the comparability category. Therefore, a maximum of nine stars could have been given for each study.

### Statistical analysis and quality evaluation of the evidence

Statistical analysis was performed using Review Manager software (version 5.3). We used the Mantel Haenszel method for all the outcomes in our meta-analysis because the outcomes were all dichotomous. The pooled outcomes were calculated using the odds ratio (OR) with 95% confidence intervals (CI). Heterogeneity was measured using I^2^ statistics (I^2^ < 50% was regarded as homogeneity) and a fixed-effects model was used in this study. P values less than 0.05 were considered statistically significant. Funnel plots were drawn using the software to assess publication bias. Sensitivity analysis was performed after excluding the studies with a small sample size (the sample size in the exposure group was less than or equal to 30). We used the grading of recommendations assessment, development, and evaluation (GRADE) system to evaluate the quality of evidence for the results [12]. Four grades were included in this working group grades of evidence: high quality, moderate quality, low quality, and very low quality.

## Results

### Literature search and study selection

The detailed search strategy is shown in the S1 Table. Fig 1 shows the process of study selection. A total of 971 studies were found in the three databases (PubMed = 191, Embase = 769, and CENTRAL = 11). No additional records were identified through other sources. After duplicates were removed, 850 records remained; however, 820 records were further removed after reading the titles and abstracts. Thirty full-text articles were read in detail for eligibility and 18 articles were excluded for various reasons. Finally, 12 studies were included in this meta-analysis [6–8,13–21].

### Study characteristics and data extraction

The characteristics of the 12 included studies are shown in Table 1. The publication year ranged from 1984 to 2016. Study areas included the United States, Japan, Europe, Israel, Taiwan, Denmark, and France. Case–control studies, retrospective cohort studies, and prospective cohort studies were included in this meta-analysis. The study periods and participants for each individual study are shown in Table 1. Tables 2 and 3 show the baseline data extracted from each study. The exposure groups included “MMI/CMZ,” “PTU,” or “MMI/CMZ and PTU.” Three studies involved a sample size in the exposure group that was less than or equal to 30 [16, 20, 21]. The definitions of “No ATD group” and “Control group” were slightly different in each included study (see Tables 2 and 3). Two studies involved exposure before pregnancy [18, 19] and another 10 studies involved exposure only during pregnancy. Seven studies involved only major congenital abnormalities [6–8, 13–15, 17] and two studies involved all birth defects [18, 19].

**Table 1:** Characteristics of the 12 included studies in this meta-analysis.

First author /Publication year	Study region	Study type	Study period	Participants
**Momotani 1984**	Japan	Case-control study	August 1965 to May 1980	643 neonates from mothers with Graves’ disease
**Wing 1994**	Unite State	Retrospective cohort study	1974 to 1990	185 pregnant patients with a history or diagnosis of hyperthyroidism
**Gianantonio 2001**	Europe	Prospective cohort study	NA	241 pregnancy women because of MMI exposure, and compared them with those of 1,089 pregnant women because of exposure to nonteratogenic drugs
**Lian 2005**	China	Retrospective cohort study	1 January 1983to 31 December 2003	100 cases of pregnancy with hyperthyroidism patients and 101 cases of newborn
**Rosenfeld 2009**	Israel	Prospective cohort study	1994 to 2004	PTU-exposed pregnancies of women and women exposed to nonteratogens
**Clementi 2010**	Unite State	Case-control study	1990 to 2004	18,131 cases with malformations and reported first-trimester exposure to medication
**Chen 2011**	Taiwan	Case-control study	1 January 2005 to 31 December 2005	A total of 2830 mothers with hyperthyroidism and 14150age-matched randomly selected mothers without hyperthyroidism
**Yoshihara 2012**	Japan	Retrospective cohort study	1 January 1999 to 31 December 2010	Women with Graves’ disease who became pregnant
**Andersen 2013**	Denmark	Retrospective cohort study	1996 to 2008	817093 children live-born
**Korelitz 2013**	Unite State	Retrospective cohort study	2005 to 2009	Women aged 15–44 years, enrolled for at least 2 years, and who had a pregnancy during the study period
**Lo 2015**	Unite State	Retrospective cohort study	1 January 1996 to 31 December 2010	All pregnancies resulting in a live birth, among women age 15–49 years at the time of delivery
**Hawken 2016**	France	Retrospective cohort study	2005 to 2012	Ninety-five pregnancies

Abbreviations are as follows: MMI, methimazole; PTU, propylthiouracil; NA, not available.

**Table 2:** Data extraction from the 12 included studies in this meta-analysis.

First author /Publication year	Exposure groups	No ATD group	Control group	Duration of exposure	Type of congenital malformations
**Momotani 1984**	MMI(2/117)	NA	Euthyroid and no MMI(1/350)	During the first trimester of pregnancy	Major malformations
**Wing 1994**	MMI(1/36) PTU(3/99)	No medications (1/43)	NA	During pregnancy	Major congenital malformations
**Gianantonio 2001**	MMI/CMZ(8/241)	Pregnant women exposure to nonteratogenic drugs (23/1089)	NA	During pregnancy	Major malformations
**Lian 2005**	MMI(5/12) PTU(1/28)	Hyperthyroidism and no ATD (1/61)	NA	During the first trimester of pregnancy	NA (Neonatal congenital malformations)
**Rosenfeld 2009**	PTU(1/80)	NA	Women exposed to nonteratogenic agents (34/1066)	Between the 4^th^ and 13^th^ gestational week	Major anomalies
**Clementi 2010**	MMI/CMZ(16/80) PTU(10/47)	NA	NA	During the first trimester of pregnancy	Major birth defect
**Chen 2011**	MMI(0/73) PTU(5/630)	Women with hyperthyroidism and not receiving ATD (15/2127)	Women in the comparison group, no hyperthyroidism and no ATD (92/14150)	During pregnancy	Major congenital anomalies
**Yoshihara 2012**	MMI(50/1426) PTU(26/1578)	Graves’ disease without medicine(40/2065)	NA	During the first trimester of pregnancy	Major malformations

Abbreviations are as follows: MMI, methimazole; CMZ, carbimazole; PTU, propylthiouracil; ATD, antithyroid drugs; NA, not available; /, or; &, and.

**Table 3:** Data extraction from the 12 included studies in this meta-analysis.

First author /Publication year	Exposure groups	No ATD group	Control group	Duration of exposure	Type of congenital malformations
**Andersen 2013**	MMI/CMZ(100/1097) PTU(45/564) MMI/CMZ &PTU(16/159)	ATD use, but not in pregnancy (190/3543)	Nonexposed, never ATD use (45982/811730)	6 months before pregnancy to the end of the 10^th^ gestational week	All birth defects
**Korelitz 2013**	MMI(6/108) PTU(66/915) MMI&PTU(14/126)	Thyrotoxicosis before/during/after pregnancy and no ATD(390/5932)	No thyrotoxicosis (37351/634858)	Over the 6 months before or during the pregnancy	Any congenital defect
**Lo 2015**	MMI(1/30) PTU(15/507) MMI&PTU(2/49)	Thyrotoxicosis diagnosis, no gestational ATD(52/1171)	NA	During pregnancy	NA (Congenital malformations)
**Hawken 2016**	CMZ(4/19) PTU(0/7)	NA	NA	During the first trimester of pregnancy	NA (Congenital malformation)

Abbreviations are as follows: MMI, methimazole; CMZ, carbimazole; PTU, propylthiouracil; ATD, antithyroid drugs; NA, not available; /, or; &, and.

### Quality assessment of the included studies

We performed a quality assessment of the 12 included studies using the NOS system, which is suitable for case–control studies and cohort studies [11]. The details of the star template are shown in the S2 Table. We found that the ascertainment of exposure and the assessment of outcome were unclear in the Gianantonio study [15]. The exposed cohort was not representative in Lian’s study because only newborn cases delivered in the Peking Union Medical College Hospital were included [16]. There was not a “No ATD group” or “Control group” in Hawken’s study [21]; therefore, no star was given for the “selection of non-exposure cohort” or “comparability of cohorts based on the design or analysis” for this study. Therefore, the three cohort studies were given a total of six stars [15, 16, 21]. “No-Response Rate” was not mentioned in any case–control study included in this study [6, 7, 13]. The ascertainment of exposure was unclear in Clementi’s study [6]. Therefore, a total of seven stars were given to the three case–control studies. Table 4 illustrates the total star template for each individual study. The results indicated that most of the data in this meta-analysis were from studies with a relatively high quality.

**Table 4:** The Newcastle-Ottawa Scale (NOS) quality assessment of the included studies in this meta-analysis.

**Cohort Star Template**				
**Study**	**Selection (4)**	**Comparability (2)**	**Outcome (3)**	**Total (9)**
**Wing**	☆☆☆☆	☆	☆☆☆	8
**Gianantonio**	☆☆☆	☆	☆☆	6
**Lian**	☆☆☆		☆☆☆	6
**Rosenfeld**	☆☆☆☆	☆	☆☆	7
**Yoshihara**	☆☆☆☆	☆	☆☆☆	8
**Andersen**	☆☆☆☆	☆☆	☆☆☆	9
**Korelitz**	☆☆☆☆	☆	☆☆☆	8
**Lo**	☆☆☆☆	☆	☆☆☆	8
**Hawken**	☆☆☆		☆☆☆	6
**Case-Control Star Template**				
**Study**	**Selection (4)**	**Comparability (2)**	**Exposure (3)**	**Total (9)**
**Momotani**	☆☆☆☆	☆	☆☆	7
**Clementi**	☆☆☆☆	☆☆	☆	7
**Chen**	☆☆☆☆	☆	☆☆	7

### Meta-analysis for the risk of neonatal congenital malformation between different groups

**Fig 2:**
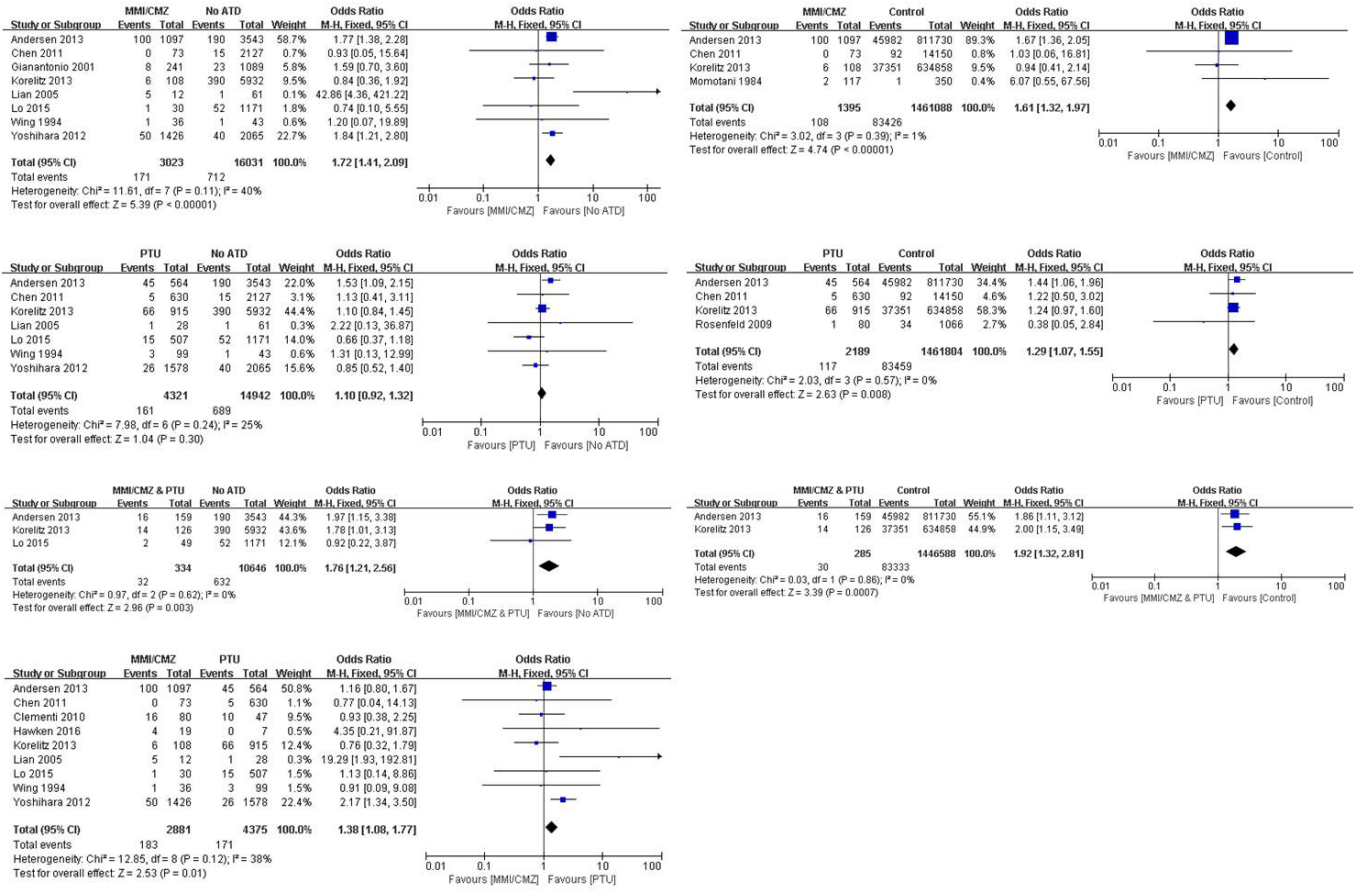
Risk of neonatal congenital malformations between different groups.

As shown in Fig 2, eight studies provided a comparison between an “MMI/CMZ” exposure group and a “No ATD” group and included a total of 19054 participants. The results indicated that the risk of neonatal congenital malformation was significantly lower in the “No ATD” group than in the “MMI/CMZ” group (OR 1.72; 95%CI 1.41 to 2.09; P < 0.00001). Four studies offered a comparison between “MMI/CMZ” and “Control” and included a total of 1462483 participants. Similar to the above results, groups exposed to MMI/CMZ had a significantly increased risk of congenital malformations compared to “Control” groups (OR 1.61; 95%CI 1.32 to 1.97; P < 0.00001). Seven studies provided a comparison between PTU exposure and “No ATD” exposure and no differences were observed between the two groups (OR 1.10; 95%CI 0.92 to 1.32; P = 0.30). In contrast, the risk of neonatal congenital malformations was significantly increased in groups exposed to PTU when compared to a “Control” group (OR 1.29; 95%CI 1.07 to 1.55; P = 0.008). Three trials reported exposure in pregnant women to “MMI/CMZ & PTU” and “No ATD” and included a total of 10980 participants. The risk of neonatal congenital malformations in mothers exposed to “MMI/CMZ & PTU” was significant higher than that in the “No ATD” groups (OR 1.76; 95%CI 1.21 to 2.56; P = 0.003). Two studies involved exposure in pregnant women to “MMI/CMZ & PTU” and “Control.” The results indicated that the risk of neonatal congenital malformations in the combination exposure group was significantly higher than that in the “Control” group (OR 1.92; 95%CI 1.32 to 2.81; P = 0.0007). Nine studies compared exposure to MMI/CMZ and PTU (2881 participants in the MMI/CMZ group; 4375 participants in the PTU group) to evaluate the risk of neonatal congenital malformations. The results indicated that exposure to MMI/CMZ significantly increased the risk of neonatal congenital malformations compared to exposure to PTU (OR 1.38; 95%CI 1.08 to 1.77; P = 0.01).

**Fig 3:**
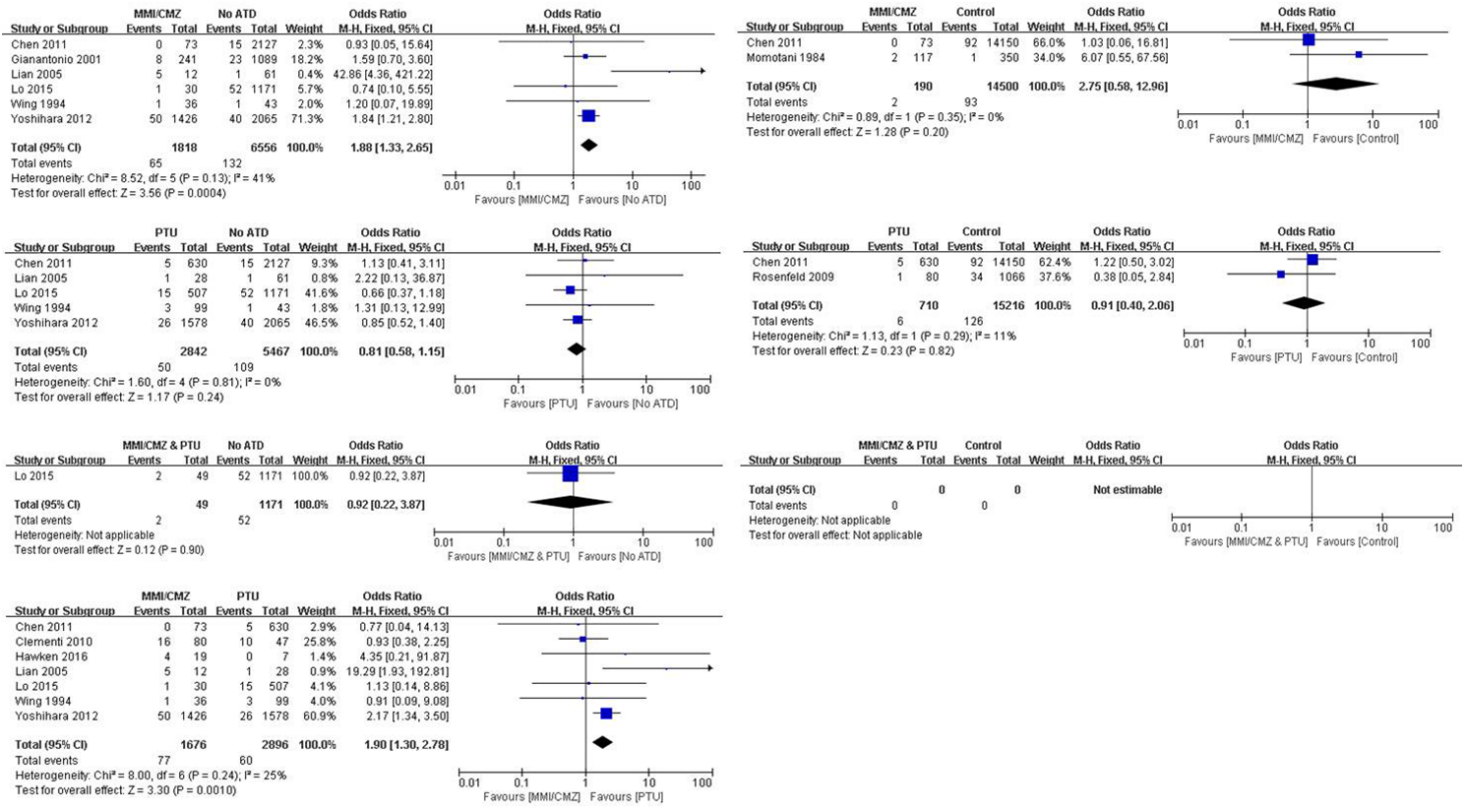
Risk of neonatal congenital malformations between different groups with exposure only during pregnancy.

Fig 3 illustrates the results after removing two studies that involved exposure before pregnancy. The results indicated that groups exposed to MMI/CMZ had a significantly increased risk of neonatal congenital malformations compared to “No ATD” groups (OR 1.88; 95%CI 1.33 to 2.65; P = 0.0004). However, no differences were found between the “MMI/CMZ” and “Control” groups (OR 2.75; 95%CI 0.58 to 12.96; P = 0.20). No differences were found between PTU exposure and “No ATD” or “Control” with respect to the risk of neonatal congenital malformations (OR 0.81; 95%CI 0.58 to 1.15; P = 0.24; OR 0.91; 95%CI 0.40 to 2.06; P = 0.82). Only one study involved “MMI/CMZ & PTU” exposure and “No ATD.” No study reported a comparison between “MMI/CMZ & PTU” exposure and a “Control” group. Similar to the 12-study meta-analysis, those exposed to PTU had a significantly lower risk of neonatal congenital malformations compared to those exposed to MMI/CMZ (OR 1.90; 95%CI 1.30 to 2.78; P = 0.001).

### Sensitivity analysis and heterogeneity test

Heterogeneity across studies was determined and sensitivity analyses were conducted. Funnel plots are shown in the S1 and S2 Figs and indicated that the three studies that involved a small sample size (sample size in exposure groups was less than or equal to 30) were the major source of heterogeneity across the studies [16, 20, 21]. Therefore, sensitivity analyses were performed after excluding those three studies. As shown in Table 5, treatment with MMI/CMZ significantly increased the risk of neonatal congenital malformations compared to “No ATD” (OR 1.68; 95%CI 1.37 to 2.05; P < 0.00001), which was consistent with the previous results. However, I^2^ decreased from 40% to 0%. The risk of neonatal congenital malformations remained not different between PTU and “No ATD” (OR 1.17; 95%CI 0.97 to 1.42; P = 0.11) and I^2^ decreased from 25% to 5%. The risk of neonatal congenital malformations appeared higher in patients exposed to MMI/CMZ than in those exposed to PTU (OR 1.31; 95%CI 1.01 to 1.69; P = 0.04), which was consistent with the previous results; however, I^2^ decreased from 38% to 29%.

**Table 5:** The summary outcomes of this meta-analysis (including those of sensitivity analysis).

**All the 12 included studies (12 studies)**	**MMI/CMZ vs. No ATD**	**MMI/CMZ vs. Control**	**PTU vs. No ATD**	**PTU vs. Control**	**MMI/CMZ &PTU vs. No ATD**	**MMI/CMZ &PTU vs. Control**	**MMI/CMZ vs. PTU**
No. of the included studies	8	4	7	4	3	2	9
Participants	19054	1462483	19263	1463993	10980	1446873	7256
Odds Ratio [95%CI]	1.72[1.41, 2.09]	1.61[1.32, 1.97]	1.10[0.92, 1.32]	1.29[1.07, 1.55]	1.76[1.21, 2.56]	1.92[1.32, 2.81]	1.38[1.08, 1.77]
I^2^	40%	1%	25%	0%	0%	0%	38%
P	<0.00001	<0.00001	0.30	0.008	0.003	0.0007	0.01
**Excluded studies with sample size less than or equal to 30 (9 studies)**	**MMI/CMZ vs. No ATD**	**MMI/CMZ vs. Control**	**PTU vs. No ATD**	**PTU vs. Control**	**MMI/CMZ &PTU vs. No ATD**	**MMI/CMZ &PTU vs. Control**	**MMI/CMZ vs. PTU**
No. of the included studies	6	4	5	4	2	2	6
Participants	17780	1462483	17496	1463993	9760	1446873	6653
Odds Ratio [95%CI]	1.68[1.37, 2.05]	1.61[1.32, 1.97]	1.17[0.97, 1.42]	1.29[1.07, 1.55]	1.88[1.27, 2.77]	1.92[1.32, 2.81]	1.31[1.01, 1.69]
I^2^	0%	1%	5%	0%	0%	0%	29%
P	<0.00001	<0.00001	0.11	0.008	0.002	0.0007	0.04
**Only major congenital malformations (7 studies)**	**MMI/CMZ vs. No ATD**	**MMI/CMZ vs. Control**	**PTU vs. No ATD**	**PTU vs. Control**	**MMI/CMZ &PTU vs. No ATD**	**MMI/CMZ &PTU vs. Control**	**MMI/CMZ vs. PTU**
No. of the included studies	4	2	3	2	0	0	4
Participants	7100	14690	6542	15926	0	0	3969
Odds Ratio [95%CI]	1.75[1.22, 2.53]	2.75[0.58, 12.96]	0.91[0.59, 1.41]	0.91[0.40, 2.06]	—	—	1.73[1.15, 2.59]
I^2^	0%	0%	0%	11%	—	—	11%
P	0.003	0.20	0.67	0.82	—	—	0.008

Abbreviations are as follows: CI, confidence interval; I^2^, heterogeneity; P, p value; MMI, methimazole; CMZ, carbimazole; PTU, propylthiouracil; ATD, antithyroid drugs; vs., versus; /, or; &, and.

Sensitivity analyses were also performed in the 10 studies with exposure only during pregnancy. Table 6 illustrates the results of the sensitivity analyses. We found that MMI/CMZ significantly increased the risk of neonatal congenital malformations compared to “No ATD” (OR 1.75; 95%CI 1.22 to 2.53; P = 0.003) and I^2^ decreased from 41% to 0%. In addition, MMI/CMZ significantly increased the risk of neonatal congenital malformations compared to PTU (OR 1.73; 95%CI 1.15 to 2.59; P = 0.008) and I^2^ decreased from 25% to 11%.

**Table 6:** The summary outcomes of this meta-analysis with exposure only during pregnancy. (including those of sensitivity analysis).

**All the 10 included studies (10 studies)**	**MMI/CMZ vs. No ATD**	**MMI/CMZ vs. Control**	**PTU vs. No ATD**	**PTU vs. Control**	**MMI/CMZ &PTU vs. No ATD**	**MMI/CMZ &PTU vs. Control**	**MMI/CMZ vs. PTU**
No. of the included studies	6	2	5	2	1	0	7
Participants	8374	14690	8309	15926	1220	0	4572
Odds Ratio [95%CI]	1.88[1.33, 2.65]	2.75[0.58, 12.96]	0.81[0.58, 1.15]	0.91[0.40, 2.06]	0.92[0.22, 3.87]	—	1.90[1.30, 2.78]
I^2^	41%	0%	0%	11%	—	—	25%
P	0.0004	0.20	0.24	0.82	0.90	—	0.001
**Excluded studies with sample size less than or equal to 30 (7 studies)**	**MMI/CMZ vs. No ATD**	**MMI/CMZ vs. Control**	**PTU vs. No ATD**	**PTU vs. Control**	**MMI/CMZ &PTU vs. No ATD**	**MMI/CMZ &PTU vs. Control**	**MMI/CMZ vs. PTU**
No. of the included studies	4	2	3	2	0	0	4
Participants	7100	14690	6542	15926	0	0	3969
Odds Ratio [95%CI]	1.75[1.22, 2.53]	2.75[0.58, 12,96]	0.91[0.59, 1.41]	0.91[0.40, 2.06]	—	—	1.73[1.15, 2.59]
I^2^	0%	0%	0%	11%	—	—	11%
P	0.003	0.20	0.67	0.82	—	—	0.008
**Only major congenital malformations (7 studies)**	**MMI/CMZ vs. No ATD**	**MMI/CMZ vs. Control**	**PTU vs. No ATD**	**PTU vs. Control**	**MMI/CMZ &PTU vs. No ATD**	**MMI/CMZ &PTU vs. Control**	**MMI/CMZ vs. PTU**
No. of the included studies	4	2	3	2	0	0	4
Participants	7100	14690	6542	15926	0	0	3969
Odds Ratio [95%CI]	1.75[1.22, 2.53]	2.75[0.58, 12,96]	0.91[0.59, 1.41]	0.91[0.40, 2.06]	—	—	1.73[1.15, 2.59]
I^2^	0%	0%	0%	11%	—	—	11%
P	0.003	0.20	0.67	0.82	—	—	0.008

Abbreviations are as follows: CI, confidence interval; I^2^, heterogeneity; P, p value; MMI, methimazole; CMZ, carbimazole; PTU, propylthiouracil; ATD, antithyroid drugs; vs., versus; /, or; &, and.

### Other outcomes—Risk of major neonatal congenital malformations

Seven studies provided the type of congenital malformation named as “major congenital malformation.” Therefore, we performed a meta-analysis that included only the studies that evaluated major neonatal congenital malformations. As shown in Table 5, exposure to MMI/CMZ significantly increased the risk of major neonatal congenital malformations compared to “No ATD” exposure (OR 1.75; 95%CI 1.22 to 2.53; P = 0.003), which was consistent with the previous results. The risk of major neonatal malformations was significantly less in those exposed to PTU compared to that in those exposed to MMI/CMZ (OR 1.73; 95%CI 1.15 to 2.59; P = 0.008). In contrast, no differences were found in the other five comparisons. The comparison of the risk of major neonatal congenital malformations between the different groups after removing two studies involving exposure before pregnancy is shown in Table 6. Consistent with the earlier results, statistical significant differences were only found in the comparisons between exposure to MMI/CMZ and “No ATD” (OR 1.75; 95%CI 1.22 to 2.53; P = 0.003) and exposure to PTU and exposure to MMI/CMZ (OR 1.73; 95%CI 1.15 to 2.59; P = 0.008).

Quality assessment of the evidence for all the results (GRADE) is shown in Tables 7–10. The results ranged from very low to high according to the evaluation criteria.

**Table 7:** GRADE for outcomes of this meta-analysis.

Outcomes	No of Participants (studies)	Quality of the evidence (GRADE)	Relative effect (95% CI)	Anticipated absolute effects	
**MMI/CMZ VS No ATD**	19054(8 studies^1^)	⊕⊝⊝⊝ **VERY LOW** due to risk of bias, inconsistency, imprecision	**OR 1.72** (1.41 to 2.09)	**Risk with No ATD**	**Risk difference with MMI/CMZ** (95% CI)
				**Study population**	
				**44 per 1000**	**30 more per 1000**(from 17 more to 44 more)
				**Moderate**	
				**22 per 1000**	**15 more per 1000**(from 9 more to 23 more)
**MMI/CMZ VS No Control**	1462483 (4 studies^1^)	⊕⊕⊝⊝ **LOW** due to inconsistency, large effect	**OR 1.61** (1.32 to 1.97)	**Risk with Control**	**Risk difference with** MMI/CMZ (95% CI)
				**Study population**	
				**57 per 1000**	**32 more per 1000**(from 17 more to 49 more)
				**Moderate**	
				**32 per 1000**	**19 more per 1000**(from 10 more to 29 more)
**PTU VS No ATD**	19263 (7 studies^1^)	⊕⊝⊝⊝ **VERY LOW** due to risk of bias, imprecision	**OR 1.1** (0.92 to 1.32)	**Risk with No ATD**	**Risk difference with PTU** (95% CI)
				**Study population**	
				**46 per 1000**	**4 more per 1000**(from 4 fewer to 14 more)
				**Moderate**	
				**23 per 1000**	**2 more per 1000**(from 2 fewer to 7 more)

The basis for the **assumed risk** (e.g. the median control group risk across studies) is provided in footnotes. The **corresponding risk** (and its 95% CI) is based on the assumed risk in the comparison group and the **relative effect** of the intervention (and its 95% CI).

**GRADE Working Group grades of evidence: High quality:** Further research is very unlikely to change our confidence in the estimate of effect. **Moderate quality:** Further research is likely to have an important impact on our confidence in the estimate of effect and may change the estimate. **Low quality:** Further research is very likely to have an important impact on our confidence in the estimate of effect and is likely to change the estimate.**Very low quality:** We are very uncertain about the estimate.

^1^ case-control and other study designs together.

**Abbreviations are as follows:** GRADE,Grading of Recommendations Assessment, Development, and Evaluations; MMI, methimazole; CMZ, carbimazole; PTU, propylthiouracil; ATD, antithyroid drugs; CI, Confidence interval; OR, Odds ratio; /, or.

**Table 8:** GRADE for outcomes of this meta-analysis.

Outcomes	No of Participants (studies)	Quality of the evidence (GRADE)	Relative effect (95% CI)	Anticipated absolute effects	
**PTU VS Control**	1463993 (4 studies^1^)	⊕⊕⊕⊕ **HIGH** due to large effect	**OR 1.29** (1.07 to 1.55)	**Risk with Control**	**Risk difference with PTU** (95% CI)
				**Study population**	
				**57 per 1000**	**15 more per 1000**(from 4 more to 29 more)
				**Moderate**	
				**44 per 1000**	**12 more per 1000**(from 3 more to 23 more)
**MMI/CMZ & PTU VS No ATD**	10980 (3 studies)	⊕⊕⊕⊝ **MODERATE** due to imprecision, large effect	**OR 1.76** (1.21 to 2.56)	**Risk with No ATD**	**Risk difference with MMI/CMZ & PTU** (95% CI)
				**Study population**	
				**59 per 1000**	**41 more per 1000**(from 12 more to 80 more)
				**Moderate**	
				**54 per 1000**	**37 more per 1000**(from 11 more to 73 more)
**MMI/CMZ & PTU VS Control**	1446873 (2 studies)	⊕⊕⊕⊕ **HIGH** due to large effect	**OR 1.92** (1.32 to 2.81)	**Risk with Control**	**Risk difference with MMI/CMZ & PTU** (95% CI)
				**Study population**	
				**58 per 1000**	**47 more per 1000**(from 17 more to 89 more)
				**Moderate**	
				**58 per 1000**	**48 more per 1000**(from 17 more to 89 more)
**MMI/CMZ VS PTU**	7256 (9 studies^1^)	⊕⊝⊝⊝ **VERY LOW** due to risk of bias, inconsistency, imprecision	**OR 1.38** (1.08 to 1.77)	**Risk with PTU**	**Risk difference with MMI/CMZ** (95% CI)
				**Study population**	
				**39 per 1000**	**14 more per 1000**(from 3 more to 28 more)
				**Moderate**	
				**30 per 1000**	**11 more per 1000**(from 2 more to 22 more)

The basis for the **assumed risk** (e.g. the median control group risk across studies) is provided in footnotes. The **corresponding risk** (and its 95% CI) is based on the assumed risk in the comparison group and the **relative effect** of the intervention (and its 95% CI).

**GRADE Working Group grades of evidence: High quality:** Further research is very unlikely to change our confidence in the estimate of effect. **Moderate quality:** Further research is likely to have an important impact on our confidence in the estimate of effect and may change the estimate. **Low quality:** Further research is very likely to have an important impact on our confidence in the estimate of effect and is likely to change the estimate.**Very low quality:** We are very uncertain about the estimate.

^1^ case-control and other study designs together.

**Abbreviations are as follows:** GRADE,Grading of Recommendations Assessment, Development, and Evaluations; MMI, methimazole; CMZ, carbimazole; PTU, propylthiouracil; ATD, antithyroid drugs; CI, Confidence interval; OR, Odds ratio; /, or; &, and.

**Table 9:** GRADE for outcomes of this meta-analysis with exposure only during pregnancy.

Outcomes	No of Participants (studies)	Quality of the evidence (GRADE)	Relative effect (95% CI)	Anticipated absolute effects	
**MMI/CMZ VS No ATD**	8374 (6 studies^1^)	⊕⊝⊝⊝ **VERY LOW** due to risk of bias, inconsistency, imprecision	**OR 1.88** (1.33 to 2.65)	**Risk with No ATD**	**Risk difference with MMI/CMZ** (95% CI)
				**Study population**	
				**20 per 1000**	**17 more per 1000** (from 6 more to 32 more)
				**Moderate**	
				**20 per 1000**	**17 more per 1000**(from 6 more to 31 more)
**MMI/CMZ VS No Control**	14690 (2 studies^2^)	⊕⊕⊕⊕ **HIGH** due to large effect	**OR 2.75** (0.58 to 12.96)	**Risk with Control**	Risk difference with MMI/CMZ (95% CI)
				**Study population**	
				**6 per 1000**	**11 more per 1000**(from 3 fewer to 71 more)
				**Moderate**	
				**5 per 1000**	**9 more per 1000**(from 2 fewer to 56 more)
**PTU VS No ATD**	8309 (5 studies^1^)	⊕⊕⊝⊝ **LOW** due to risk of bias, imprecision, large effect	**OR 0.81** (0.58 to 1.15)	**Risk with No ATD**	Risk difference with PTU (95% CI)
				**Study population**	
				**20 per 1000**	**4 fewer per 1000**(from 8 fewer to 3 more)
				**Moderate**	
				**19 per 1000**	**4 fewer per 1000**(from 8 fewer to 3 more)

The basis for the **assumed risk** (e.g. the median control group risk across studies) is provided in footnotes. The **corresponding risk** (and its 95% CI) is based on the assumed risk in the comparison group and the **relative effect** of the intervention (and its 95% CI).

**GRADE Working Group grades of evidence: High quality:** Further research is very unlikely to change our confidence in the estimate of effect. **Moderate quality:** Further research is likely to have an important impact on our confidence in the estimate of effect and may change the estimate. **Low quality:** Further research is very likely to have an important impact on our confidence in the estimate of effect and is likely to change the estimate. **Very low quality:** We are very uncertain about the estimate.

^1^ case-control and other study designs together.

^2^ case-control.

**Abbreviations are as follows:** GRADE,Grading of Recommendations Assessment, Development, and Evaluations; MMI, methimazole; CMZ, carbimazole; PTU, propylthiouracil; ATD, antithyroid drugs; CI, Confidence interval; OR, Odds ratio; /, or.

**Table 10:** GRADE for outcomes of this meta-analysis with exposure only during pregnancy.

Outcomes	No of Participants (studies)	Quality of the evidence (GRADE)	Relative effect (95% CI)	Anticipated absolute effects	
**PTU VS Control**	15926 (2 studies^1^)	⊕⊕⊕⊝ **MODERATE** due to large effect	**OR 0.91** (0.4 to 2.06)	**Risk with Control**	**Risk difference with PTU** (95% CI)
				**Study population**	
				**8 per 1000**	**1 fewer per 1000**(from 5 fewer to 9 more)
				**Moderate**	
				**19 per 1000**	**2 fewer per 1000**(from 11 fewer to 19 more)
**MMI/CMZ & PTU VS No ATD**	1220 (1 study)	⊕⊝⊝⊝ **VERY LOW** due to imprecision	**OR 0.92** (0.22 to 3.87)	**Risk with No ATD**	**Risk difference with MMI/CMZ & PTU** (95% CI)
				**Study population**	
				**44 per 1000**	**3 fewer per 1000**(from 34 fewer to 108 more)
				**Moderate**	
				**44 per 1000**	**3 fewer per 1000**(from 34 fewer to 107 more)
**MMI/CMZ VS PTU**	4572 (7 studies^1^)	⊕⊝⊝⊝ **VERY LOW** due to risk of bias, inconsistency, imprecision	**OR 1.9** (1.3 to 2.78)	**Risk with PTU**	**Risk difference with MMI/CMZ** (95% CI)
				**Study population**	
				**21 per 1000**	**18 more per 1000**(from 6 more to 35 more)
				**Moderate**	
				**30 per 1000**	**26 more per 1000**(from 9 more to 49 more)

The basis for the **assumed risk** (e.g. the median control group risk across studies) is provided in footnotes. The **corresponding risk** (and its 95% CI) is based on the assumed risk in the comparison group and the **relative effect** of the intervention (and its 95% CI).

**GRADE Working Group grades of evidence: High quality:** Further research is very unlikely to change our confidence in the estimate of effect. **Moderate quality:** Further research is likely to have an important impact on our confidence in the estimate of effect and may change the estimate. **Low quality:** Further research is very likely to have an important impact on our confidence in the estimate of effect and is likely to change the estimate. **Very low quality:** We are very uncertain about the estimate.

^1^ case-control and other study designs together.

**Abbreviations are as follows:** GRADE,Grading of Recommendations Assessment, Development, and Evaluations; MMI, methimazole; CMZ, carbimazole; PTU, propylthiouracil; ATD, antithyroid drugs; CI, Confidence interval; OR, Odds ratio; /, or; &, and.

## Discussion

We divided our meta-analysis into two parts. The first part evaluated the outcomes of the 12 included studies. Exposure to MMI/CMZ during pregnancy significantly increased the risk of neonatal congenital malformations compared to “No ATD” groups and “Control” groups (the specific definition of “No ATD” and “Control” in each individual study are presented in Tables 2 and 3). These results were consistent with those of previous studies [5, 9]. The risk of neonatal congenital malformations appeared to be higher in groups exposed to PTU compared to “Control” groups. However, no difference was observed between groups exposed to PTU and “No ATD” groups, which was different from the conclusion described by Li et al. [5]. This result indicated that neonatal congenital malformations that occurred in groups exposed to PTU may have been due to the gestational hyperthyroidism disorders rather than the administration of PTU. Therefore, we speculated that hyperthyroid states during pregnancy were not only associated with pregnancy loss, reduced fetal growth, and thyroid storm [22], but also put the fetus at a high risk of birth defects, which may exceed that of PTU exposure.

Three studies involved a change in drug exposure from MMI/CMZ to PTU (or vice versa) during pregnancy [18–20]. A group that changed from one drug to another was defined as an “MMI/CMZ & PTU” group in our study. As expected, the risk of neonatal congenital malformation in the “MMI/CMZ & PTU” groups was significantly higher than that in the “No ATD” or “Control” groups, which further suggested that MMI/CMZ was associated with a higher risk of birth defects. This result agreed with the report in the Food and Drug Administration Adverse Event Reporting System files, in which there were 19 reports of birth defects following exposure to PTU (5% of all reports) and 40 reports following exposure to MMI (6.4% of all reports). The teratogenic effect of MMI may be associated with placental transfer [23, 24].

The essential comparison between MMI/CMZ and PTU exposure was performed using data from nine included studies. The results indicated that exposure to MMI/CMZ significantly increased the risk of neonatal congenital malformations compared to PTU exposure. This was consistent with the conclusions of the previous studies [9, 25]. The reasons for the negative findings reported by several trials may have been due to the methods used in their research [26].

The above results indicated that MMI/CMZ was not safe for treating hyperthyroidism during pregnancy and PTU was safer than MMI/CMZ with respect to the risk of neonatal congenital malformations. Sensitivity analyses were conducted after excluding three studies with a small sample size in the exposure group (less than or equal to 30), which indicated that these results were robust.

The duration of exposure was different for each individual study. Duration of exposure was described as “the first trimester of pregnancy” in five studies [6, 8, 13, 16, 21], “during pregnancy” in four studies [7, 14, 15, 20], “between the 4^th^ and 13^th^ gestational week” in one study [17], “6 months before pregnancy to the end of the 10^th^ gestational week” in one study [18], and “over the 6 months before or during the pregnancy” in one study [19]. To eliminate the influence of exposure before pregnancy, we excluded the two studies conducted by Andersen and Korelitz. Therefore, the second part of our meta-analysis evaluated the 10 included studies in which the exposure was only during pregnancy.

MMI/CMZ significantly increased the risk of neonatal congenital malformations compared to “No ATD” groups; however, no difference was observed between MMI/CMZ and “Control” groups. The study bias (n = 146283 vs. n = 14690) and the different outcomes (‘any congenital defect’ vs. ‘major congenital anomalies’) may explain the different results from the comparison between MMI/CMZ and “Control” groups after exclusion of the 2 articles with before pregnancy exposure. No differences were observed between the group exposed to PTU and the “No ATD” or “Control” groups. No differences were observed in the comparison of MMI/CMZ vs. “Control” or PTU vs. “Control” when we limited the exposure to only during pregnancy. These results suggested that uncontrolled hyperthyroidism before pregnancy maybe associated with the occurrence of neonatal congenital malformations, and therefore, control of hyperthyroidism before pregnancy is recommended. Only one study provided a comparison between a group exposed to “MMI/CMZ & PTU” and a “No ATD” group, whereas no study provided a comparison between a group exposed to “MMI/CMZ & PTU” and a “Control” group. The results of the comparison between MMI/CMZ and PTU agreed with previous results and indicated that PTU was safer than MMI/CMZ with respect to the risk of neonatal congenital malformations after we limited the exposure to only during pregnancy. Sensitivity analyses indicated the results were robust.

Generally, the incidence of birth defects diagnosed before 2 years of age was approximately 6% [27]. The first report of a birth defect following the use of MMI during pregnancy was a case of aplasia cutis [28]. Thereafter, various other types of birth defects were reported. PTU was usually associated with face and neck malformations (preauricular sinus and cysts). In comparison, MMI was usually associated with musculoskeletal, integumentary, digestive, and ocular defects [29, 30]. Combination therapy was associated with urinary system malformations [18].

In this study, we therefore performed a meta-analysis with respect to different types of neonatal congenital malformations. Seven studies described the malformations using the word “major” [6–8,13–15,17]. Lian, Lo, and Hawken described the malformations as “congenital malformations,” Andersen described the malformations as “all birth defects,” and Korelitz described the malformations as “any congenital defect.” Therefore, we further evaluated the risk of the major neonatal congenital malformations between different groups. Groups exposed to MMI/CMZ had a significantly increased risk of major neonatal congenital malformations compared to “No ATD” groups. No difference was observed between MMI/CMZ and “Control” groups. No differences were observed between groups exposed to PTU and “No ATD” or “Control” groups. Groups exposed to PTU had a lower risk of major neonatal congenital malformations compared to groups exposed to MMI/CMZ. Additionally, when we limited the exposure to only during pregnancy, we found that the results using the 10 studies were the same as those using the 12 studies. Therefore, we concluded that the risk of major neonatal congenital malformations following exposure to MMI/CMZ only during pregnancy was significantly higher than that following exposure to PTU.

### Limitations in current evidence

There were several limitations to our study. First, unpublished studies were not included in our study, which may have induced publication bias. Second, the quality assessment of the included studies suggested that not all the studies were of high quality, which may have affected the accuracy of the results. Third, the quality assessment of the evidence for the results ranged from very low to high, suggesting that the quality of the evidence needed further verification. Fourth, the exact time of exposure during pregnancy was not described in each individual trial; therefore, we cannot provide evidence for a safer administration time of ATDs during pregnancy.

## Conclusions

In this updated meta-analysis, the risk of neonatal congenital malformations after exposure to different ATDs during pregnancy was determined. In summary, for pregnant women with hyperthyroidism, no differences were observed between PTU exposure and no ATD exposure; however, PTU exposure led to less neonatal congenital malformations than MMI/CMZ exposure. Therefore, PTU was recommended during pregnancy with respect to neonatal congenital malformations. More trials are needed to confirm this conclusion in the future.

## Supporting information

S1 Table Search strategy. (PDF)

S2 Table The Newcastle-Ottawa Scale (NOS) quality assessment of the included studies in this meta-analysis (details). (PDF)

S3 Table PRISMA checklist. (PDF)

S1 Fig Funnel plot of the 12 included studies. (TIF)

S2 Fig Funnel plot of the 10 included studies involved exposure only during pregnancy. (TIF)

## Abbreviations

MMI: methimazole
CMZ: carbimazole
PTU: propylthiouracil
ATDs: antithyroid drugs
GRADE: Grading of Recommendations Assessment, Development, and Evaluations
NOS: the Newcastle-Ottawa Scale
OR: Odds ratio
95%CI: 95% confidence interval
I^2^: heterogeneity
P: p value
vs.: versus
/: or; &, and
