# Supplementary material for: Effects of methimazole and propylthiouracil exposure during pregnancy on the risk of neonatal congenital malformations: A meta-analysis
Source: PLoS One. 2017 Jul 3;12(7):e0180108. doi: 10.1371/journal.pone.0180108 (PMC5495385; doi:10.1371/journal.pone.0180108)
Supplement: S1 Table — (PDF) [file pone.0180108.s001.pdf]

Supplementary table 1. Search strategy

PubMed

#1 "Pregnancy"[Mesh] 797941
#2 (Pregnancies[Title/Abstract]) OR Gestation[Title/Abstract] 160631
#3 #1 OR #2 839598
#4 "Methimazole"[Mesh] 3017
#5 ((((((((((((((((((((((((((((((((Methyl-2-mercaptoimidazole[Title/Abstract]) OR 1 Methyl 2 mercaptoimidazole[Title/Abstract]) OR Methylmercaptoimidazole[Title/Abstract]) OR Tiamazol[Title/Abstract]) OR Thimazol[Title/Abstract]) OR Mercasolyl[Title/Abstract]) OR Mercazolyl[Title/Abstract]) OR Methymazol[Title/Abstract]) OR Merkazolil[Title/Abstract]) OR Thiamazole[Title/Abstract]) OR Mercazol[Title/Abstract]) OR Mercazole[Title/Abstract]) OR Metisol[Title/Abstract]) OR Metizol[Title/Abstract]) OR Tapazole[Title/Abstract]) OR Jones Brand of Methimazole[Title/Abstract]) OR Eli Lilly Brand of Methimazole[Title/Abstract]) OR Tirodril[Title/Abstract]) OR Estedi Brand of Methimazole[Title/Abstract]) OR Strumazol[Title/Abstract]) OR Nourypharma Brand of Methimazole[Title/Abstract]) OR Thiamazol Henning[Title/Abstract]) OR Henning, Thiamazol[Title/Abstract]) OR SanofiSynthelabo Brand of Methimazole[Title/Abstract]) OR Henning Berlin Brand of Methimazole[Title/Abstract]) OR ThiamazolHexal[Title/Abstract]) OR Hexal, Thiamazol[Title/Abstract]) OR Hexal Brand of Methimazole[Title/Abstract]) OR Thyrozol[Title/Abstract]) OR Merck Brand of Methimazole[Title/Abstract]) OR Favistan[Title/Abstract]) OR Temmler Brand of Methimazole[Title/Abstract]) OR Methizol[Title/Abstract]) OR Philopharm Brand of Methimazole[Title/Abstract]) OR Methylthiouracil[Title/Abstract] 1123
#6 #4 OR #5 3790
#7 "Propylthiouracil"[Mesh] 4045
#8 (6-Propyl-2-Thiouracil[Title/Abstract]) OR 6 Propyl2 Thiouracil[Title/Abstract] 157
#9 #7 OR #8 4103
#10 "Carbimazole"[Mesh] 1044
#11 ((((((((CarbimazoleHenning[Title/Abstract]) OR SanofiSynthelabo Brand of Carbimazole[Title/Abstract]) OR Henning Berlin 37
#12 #10 OR #11 1054
#13 "Antithyroid Agents"[Mesh] 5566
#14 ((((((((Agents, Antithyroid[Title/Abstract]) OR Thyroid Antagonists[Title/Abstract]) OR Antagonists, Thyroid[Title/Abstract]) 7735

#15 #13 OR #14 10893
#16 #6 OR #9 OR #12 OR #15 16117
#17 "Congenital Abnormalities"[Mesh] 525239
#18 ((((((((((((((((Abnormality, Congenital[Title/Abstract]) OR Congenital Abnormality[Title/Abstract]) OR Deformities[Title/Abstract]) OR Deformity[Title/Abstract]) OR Congenital Defects[Title/Abstract]) OR Congenital Defect[Title/Abstract]) OR Defect, Congenital[Title/Abstract]) OR Defects, Congenital[Title/Abstract]) OR Abnormalities, Congenital[Title/Abstract]) OR Birth Defects[Title/Abstract]) OR Birth Defect[Title/Abstract]) OR Defect, Birth[Title/Abstract]) OR Defects, Birth[Title/Abstract]) OR Congenital malformations[Title/Abstract]) OR Abnormalities-drug induced[Title/Abstract]) OR Congenital anomalies[Title/Abstract]) OR Pregnancy outcomes[Title/Abstract]) OR Neonatal outcomes[Title/Abstract] 209357
#19 #17 OR #18 627236
#20 #3 AND #16 AND #19 191

EMBASE
#1 ‘Pregnancy’/exp 668173
#2 'pregnancies':ab,ti OR 'gestation':ab,ti 207209
#3 #1 OR #2 746955
#4 'thiamazole'/exp 8320
#5 'methimazole':ab,ti OR 'methyl-2-mercaptoimidazole':ab,ti OR '1 methyl 2 mercaptoimidazole':ab,ti OR 'methylmercaptoimidazole':ab,ti OR 'tiamazol':ab,ti OR 'thimazol':ab,ti OR 'mercasolyl':ab,ti OR 'mercazolyl':ab,ti OR 'methymazol':ab,ti OR 'merkazolil':ab,ti OR 'thiamazole':ab,ti OR 'mercazol':ab,ti OR 'mercazole':ab,ti OR 'metisol':ab,ti OR 'metizol':ab,ti OR 'tapazole':ab,ti OR 'jones brand of methimazole':ab,ti OR 'elililly brand of methimazole':ab,ti OR 'tirodril':ab,ti OR 'estedi brand of methimazole':ab,ti OR 'strumazol':ab,ti OR 'nourypharma brand of methimazole':ab,ti OR 'thiamazolhenning':ab,ti OR 'henning, thiamazol':ab,ti OR 'sanofisynthelabo brand of methimazole':ab,ti OR 'henning berlin brand of methimazole':ab,ti OR 'thiamazolhexal':ab,ti OR 'hexal, thiamazol':ab,ti OR 'hexal brand of methimazole':ab,ti OR 'thyrozol':ab,ti OR 'merck brand of methimazole':ab,ti OR 'favistan':ab,ti OR 'temmler brand of methimazole':ab,ti OR 'methizol':ab,ti OR 'philopharm brand of methimazole':ab,ti OR 'methylthiouracil':ab,ti 4614
#6 #4 OR #5 9446
#7 'Propylthiouracil'/exp 9055
#8 '6-propyl-2-thiouracil':ab,ti OR '6 propyl 2 thiouracil':ab,ti 184
#9 #7 OR #8 9086
#10 'Carbimazole'/exp 3673
#11 'carbimazolehenning':ab,ti OR 'sanofisynthelabo brand of carbimazole':ab,ti

OR 'henning berlin brand of carbimazole':ab,ti OR 'neo-thyreostat':ab,ti OR 'herbrand brand of carbimazole':ab,ti OR 'neomercazole':ab,ti OR 'roche brand of carbimazole':ab,ti OR 'neo-mercazole':ab,ti OR 'neo tomizol':ab,ti OR 'tarbis brand of carbimazole':ab,ti 43

#12 #10 OR #11 3689

#13 'antithyroid agent'/exp 90479

#14 'antithyroid agents':ab,ti OR 'agents, antithyroid':ab,ti OR 'thyroid antagonists':ab,ti OR 'antagonists, thyroid':ab,ti OR 'antithyroid drugs':ab,ti OR 'drugs, antithyroid':ab,ti OR 'goitrogens':ab,ti OR 'antithyroid effect':ab,ti OR 'effect, antithyroid':ab,ti OR 'antithyroid effects':ab,ti OR 'effects, antithyroid':ab,ti 2608

#15 #13 OR #14 90922

#16 #6 OR #9 OR #12 OR #15 91404

#17 'congenital disorder'/exp 1219310

#18 'congenital abnormalities':ab,ti OR 'abnormality, congenital':ab,ti OR 'congenital abnormality':ab,ti OR 'deformities':ab,ti OR 'deformity':ab,ti OR 'congenital defects':ab,ti OR 'congenital defect':ab,ti OR 'defect, congenital':ab,ti OR 'defects, congenital':ab,ti OR 'abnormalities, congenital':ab,ti OR 'birth defects':ab,ti OR 'birth defect':ab,ti OR 'defect, birth':ab,ti OR 'defects, birth':ab,ti OR 'congenital malformations':ab,ti OR 'abnormalities-drug induced':ab,ti OR 'congenital anomalies':ab,ti OR 'pregnancy outcomes':ab,ti OR 'neonatal outcomes':ab,ti 128750

#19 #17 OR #18 1277980

#20 #3 AND #16 AND #19 769

CENTRAL (Cochrane Central Register of Controlled Trials)

#1 MeSH descriptor: [Pregnancy] explode all tree 6486

#2 ‘Pregnancies’ or ’Gestation’:ti,ab,kw (Word variations have been searched) 31799

#3 #1 OR #2 32008

#4 MeSH descriptor: [Methimazole] explode all trees 135

#5 ‘thiamazole’ or ‘Methyl-2-mercaptoimidazole’ or ‘1 Methyl 2 mercaptoimidazole’ or ‘Methylmercaptoimidazole’ or ‘Tiamazol’ or ‘Thimazol’ or ‘Mercasolyl’ or ‘Mercazolyl’ or ‘Methymazol’ or ‘Merkazolil’ or ‘Thiamazole’ or ‘Mercazol’ or ‘Mercazole’ or ‘Metisol’ or ‘Metizol’ or ‘Tapazole’ or ‘Jones Brand of Methimazole’ or ‘Eli Lilly Brand of Methimazole’ or ‘Tirodril’ or ‘Estedi Brand of Methimazole’ or ‘Strumazol’ or ‘Nourypharma Brand of Methimazole’ or ‘ThiamazolHenning’OR ‘Henning, Thiamazol’ or ‘SanofiSynthelabo Brand of Methimazole’ or ‘Henning Berlin Brand of Methimazole’ or ‘ThiamazolHexal’ or ‘Hexal, Thiamazol’ or ‘Hexal Brand of Methimazole’ or ‘Thyrozol’ or ‘Merck Brand of Methimazole’ or ‘Favistan’ or ‘Temmler Brand of Methimazole’ or ‘Methizol’ or ‘Philopharm Brand of Methimazole’ or ‘Methylthiouracil’:ti,ab,kw (Word variations have been searched) 85

#6 #4 OR #5 199
#7 MeSH descriptor: [Propylthiouracil] explode all trees 62
#8 ‘6-Propyl-2-Thiouracil’ or ‘6 Propyl 2 Thiouracil’:ti,ab,kw (Word variations have been searched) 3
#9 #7 OR #8 64
#10 MeSH descriptor: [Carbimazole] explode all trees 46
#11 ‘Carbimazole Henning’ or ‘SanofiSynthelabo Brand of Carbimazole’ or ‘Henning Berlin Brand of Carbimazole’ or ‘Neo-Thyreostat’ or ‘Herbrand Brand of Carbimazole’ or ‘Neomercazole’ or ‘Roche Brand of Carbimazole’ or ‘Neo-Mercazole’ or ‘Neo Tomizol’ or ‘Tarbis Brand of Carbimazole’:ti,ab,kw (Word variations have been searched) 0
#12 #10 OR #11 46
#13 MeSH descriptor: [Antithyroid Agents] explode all trees 178
#14 ‘Antithyroid Agent’ or ‘Agents, Antithyroid’ or ‘Thyroid Antagonists’ or ‘Antagonists, Thyroid’ or ‘Antithyroid Drugs’ or ‘Drugs, Antithyroid’ or ‘Goitrogens’ or ‘Antithyroid Effect’ or ‘Effect, Antithyroid’ or ‘Antithyroid Effects’ or ‘Effects, Antithyroid’:ti,ab,kw (Word variations have been searched) 390
#15 #13 OR #14 390
#16 #6 OR #9 OR #12 OR #15 510
#17 MeSH descriptor: [Congenital Abnormalities] explode all trees 4384
#18 ‘Congenital disorder’ or ‘Abnormality, Congenital’ or ‘Congenital Abnormality’ or ‘Deformities’ or ‘Deformity’ or ‘Congenital Defects’ or ‘Congenital Defect’ or ‘Defect, Congenital’ or ‘Defects, Congenital’ or ‘Abnormalities, Congenital’ or ‘Birth Defects’ or ‘Birth Defect’ or ‘Defect, Birth’ or ‘Defects, Birth’ or ‘Congenital malformations’ or ‘Abnormalities-drug induced’ or ‘Congenital anomalies’ or ‘Pregnancy outcomes’ or ‘Neonatal outcomes’:ti,ab,kw (Word variations have been searched) 19130
#19 #17 OR #18 22315
#20 #3 AND #16 AND #19 11(9 Trials; 2Cochrane Reviews)
