# Supplementary material for: Effects of methimazole and propylthiouracil exposure during pregnancy on the risk of neonatal congenital malformations: A meta-analysis
Source: PLoS One. 2017 Jul 3;12(7):e0180108. doi: 10.1371/journal.pone.0180108 (PMC5495385; doi:10.1371/journal.pone.0180108)
Supplement: S2 Table — (PDF) [file pone.0180108.s002.pdf]

Supplementary table 2. The Newcastle-Ottawa Scale (NOS) quality assessment of the included studies in this meta-analysis(details).

Cohort Star Template

Study	Selection of cohorts				Comparability of cohorts	Outcome		
	Representativeness of the exposed cohort	Selection of the non exposed cohort	Ascertainment of exposure	Demonstration that outcome of interest was not present at start of study	Comparability of cohorts on the basis of the design or analysis	Assessment of outcome	Was follow up long enough for outcomes to occur	Adequacy of follow up of cohorts
**Wing**	☆	☆	☆	☆	☆	☆	☆	☆
**Gianantonio**	☆	☆		☆	☆		☆	☆
**Lian**		☆	☆	☆		☆	☆	☆
**Rosenfeld**	☆	☆	☆	☆	☆	☆	☆	
**Yoshihara**	☆	☆	☆	☆	☆	☆	☆	☆
**Andersen**	☆	☆	☆	☆	☆☆	☆	☆	☆
**Korelitz**	☆	☆	☆	☆	☆	☆	☆	☆
**Lo**	☆	☆	☆	☆	☆	☆	☆	☆
**Hawken**	☆		☆	☆		☆	☆	☆

Case-Control Star Template

Study	Selection of case and controls				Comparability of cases and controls	Exposure		
	Is the case definition adequate	Representativeness of the cases	Selection of Controls	Definition of Controls	Comparability of cases and controls on the basis of the design or analysis	Ascertainment of exposure	Same method of ascertainment for casesandcontrols	Non-Response Rate
**Momotani**	☆	☆	☆	☆	☆	☆	☆	
**Clementi**	☆	☆	☆	☆	☆☆		☆	
**Chen**	☆	☆	☆	☆	☆	☆	☆	

Supplementary table 2. The Newcastle-Ottawa Scale (NOS) quality assessment of the included studies in this meta-analysis(details).

Cohort Star Template

Study	Selection of cohorts				Comparability of cohorts	Outcome		
	Representativeness of the exposed cohort	Selection of the non exposed cohort	Ascertainment of exposure	Demonstration that outcome of interest was not present at start of study	Comparability of cohorts on the basis of the design or analysis	Assessment of outcome	Was follow up long enough for outcomes to occur	Adequacy of follow up of cohorts
**Wing**	☆	☆	☆	☆	☆	☆	☆	☆
**Gianantonio**	☆	☆		☆	☆		☆	☆
**Lian**		☆	☆	☆		☆	☆	☆
**Rosenfeld**	☆	☆	☆	☆	☆	☆	☆	
**Yoshihara**	☆	☆	☆	☆	☆	☆	☆	☆
**Andersen**	☆	☆	☆	☆	☆☆	☆	☆	☆
**Korelitz**	☆	☆	☆	☆	☆	☆	☆	☆
**Lo**	☆	☆	☆	☆	☆	☆	☆	☆
**Hawken**	☆		☆	☆		☆	☆	☆

Case-Control Star Template

Study	Selection of case and controls				Comparability of cases and controls	Exposure		
	Is the case definition adequate	Representativeness of the cases	Selection of Controls	Definition of Controls	Comparability of cases and controls on the basis of the design or analysis	Ascertainment of exposure	Same method of ascertainment for casesandcontrols	Non-Response Rate
**Momotani**	☆	☆	☆	☆	☆	☆	☆	
**Clementi**	☆	☆	☆	☆	☆☆		☆	
**Chen**	☆	☆	☆	☆	☆	☆	☆	
