# Supplementary figures and images for: Effects of methimazole and propylthiouracil exposure during pregnancy on the risk of neonatal congenital malformations: A meta-analysis
Source: PLoS One. 2017 Jul 3;12(7):e0180108. doi: 10.1371/journal.pone.0180108 (PMC5495385; doi:10.1371/journal.pone.0180108)

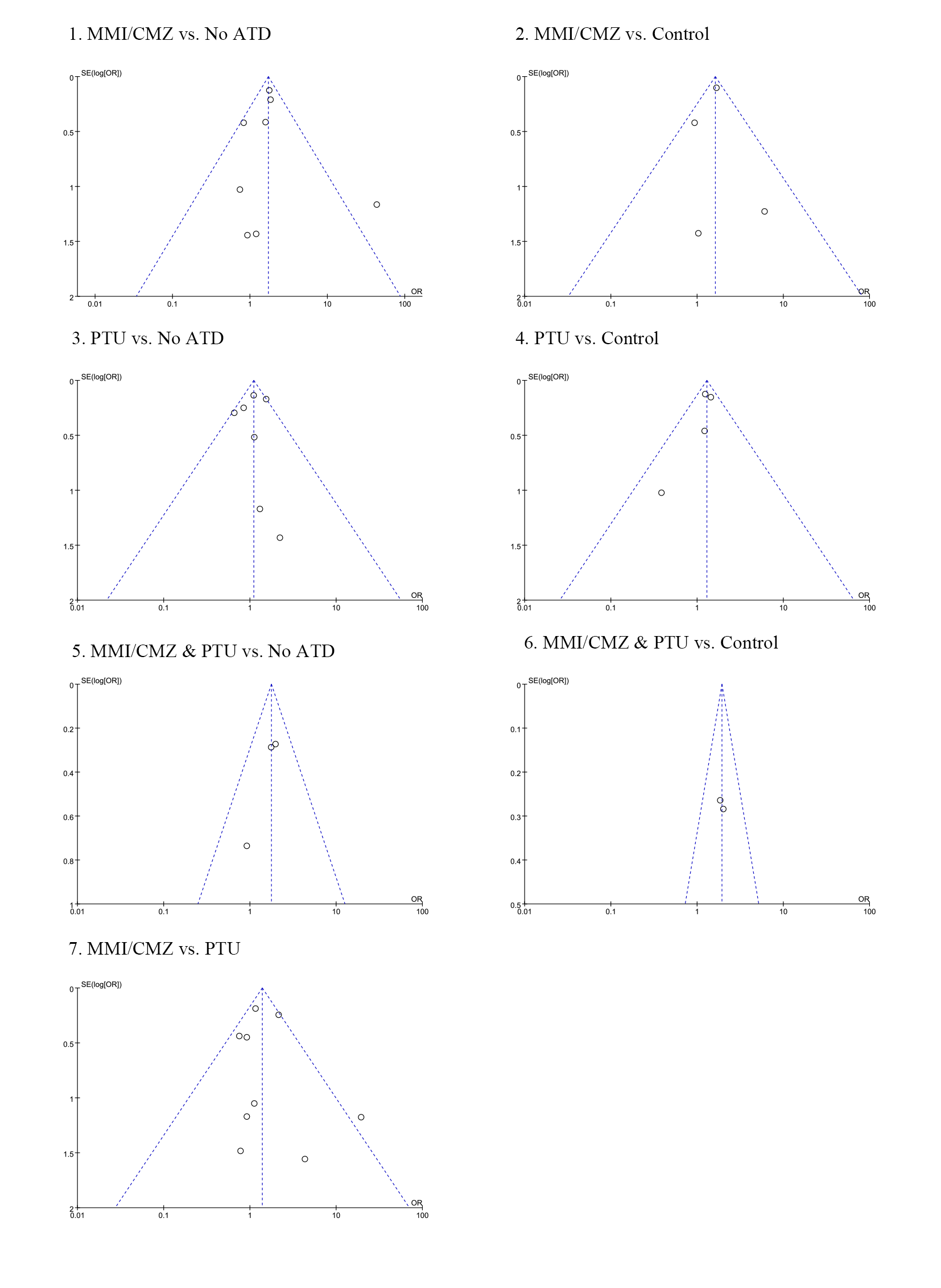

Supplement: S1 Fig — (TIF) [file pone.0180108.s004.tif]

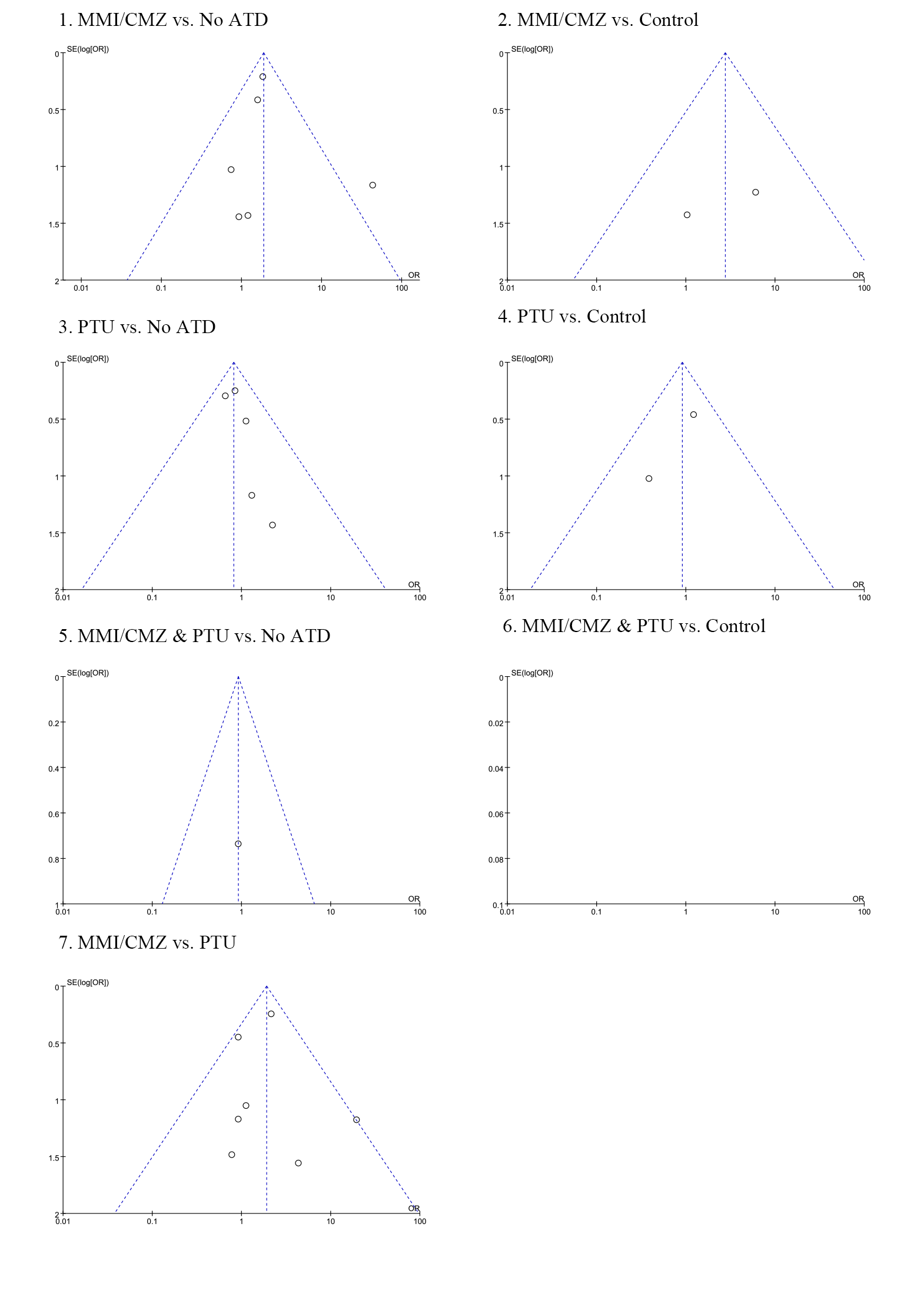

Supplement: S2 Fig — (TIF) [file pone.0180108.s005.tif]
